# Frailty in the over 65’s undergoing elective surgery (FIT-65) – a three-day study examining the prevalence of frailty in patients presenting for elective surgery

**DOI:** 10.1186/s13741-022-00272-1

**Published:** 2022-08-25

**Authors:** Sarah Harrison, David A. Harvie, Frances Wensley, Lewis Matthews, Southcoast Perioperative Audit, Research Collaboration (SPARC), William Denehan, Ciaran Barlow, Davina Ding, Dylan Green, Emma Grace, Joseph Read, Kerensa Houghton, Charlotte Towell, Neha Gupta, Oliver Cummin, Ramayee Sivasubramanian, Alex Fahmy, Andrew Cumpstey, Anna Todd, Gabor Jessica TrembickijRose, Luke Bracegirdle, Shiv Vohra, Simon Williams, Sophia Beeby, Mitul Patel, Victoria Dawe, James Collis, Chris Tyller-Veal, Sophie Ellis, Robyn Lee, Vincent McGovern, Rachel Williams, Samantha McEwan, Emma Derby, Oshine Saxena, Victoria Van Der Schyff, Fiona Kirkham, Stephanie Kirby, Charlotte Sandberg, Charlotte Philips, Rory Sharvill, Chintan Vora, Becky Sands, Becky Smart, Jack Maynard, Anthony Fung, Kate Elliot, Samuel Bhattacharjee, Siobhan Orr, Alexander Hamilton, Nicholas Stafford, Amy Greenwood, Charlie Penn, Avinash Aswath, David Massingberd-Mundy, Jessica Bailey, Miranda Davies, Michael Eddie

**Affiliations:** 1 Portsmouth Hospitals NHS Trust, Portsmouth, UK; 2 University Hospital Southampton NHS Foundation Trust, Southampton, UK; 3 The South Coast Perioperative Audit and Research Collaborative, Wessex, UK; 4 National Institute for Health Research Academic Clinical Fellow, Southampton, UK

**Keywords:** Frailty, Pre-operative risk assessment, Reported Edmonton Frail Scale, Pre-operative optimisation

## Abstract

**Background:**

Frailty increases the risk of perioperative complications, length of stay, and the need for assisted-living after discharge. As the UK population ages the number of frail patients presenting for elective surgery in the UK is likely to grow. Despite the potential benefits of early diagnosis, frailty is not uniformly screened for in UK elective surgical patients and its prevalence remains unclear. The primary aim of this study was to assess the prevalence of frailty in patients aged over 65 years undergoing elective surgery.

**Methods:**

We performed a prospective cross-sectional observational study in eight UK hospitals. Data were collected over three consecutive days with follow-up at 30 days. HRA approval was obtained (REC 20/SC/0121) and signed informed consent obtained. Participants were eligible for inclusion if they were 65 years or older and undergoing elective surgery. Pre-operative data were collected from hospital notes by anaesthetic trainees. A member of the research team blinded to the pre-operative dataset screened each participant for frailty pre-operatively using the Reported Edmonton Frail Scale (REFS). Post-operative data were collected from the notes on day of surgery and at 30 days. Participants were defined as “frail” if they scored 8 or more on the REFS.

**Results:**

Two hundred twenty eight participants were recruited during the study period of whom 218 proceeded to surgery. There were 103 females and 115 males. Median age was 75 years (interquartile range 70–80). Thirty-seven participants (17.0%) were identified as frail. Frail patients were older, had a higher ASA score, were more likely to have carers and were more likely to be anaemic or present with ECG abnormalities. There were no differences in gender, BMI, place of residence or smoking status for patients identified as frail versus non-frail. There was no difference in length-of-stay between frail and non-frail patients, although those identified as frail were less likely to be discharged to their own home.

**Conclusion:**

We found the prevalence of frailty in a mixed population of elective surgical patients aged 65 or over to be 17.0%. Furthermore, we found the REFS to be a practical tool for pre-operative frailty screening. Frail patients presented for elective surgery with modifiable co-morbidities which could have been optimised pre-operatively. Early screening could highlight frail patients, allowing time for pre-operative planning and evidence-based optimisations of comorbidities. We therefore encourage the adoption of frailty assessment as a routine part of pre-operative assessment.

## Background

The association between frailty and adverse surgical outcome is widely acknowledged (Activity 2006; Anaesthesia 2022). Frailty, defined as a ‘’distinctive health state related to the ageing process in which multiple body systems gradually lose their in-built reserves,’’ (Aucoin et al. 2020) has been shown to increase the risk of surgical complications, length of stay, and the need for assisted-living following hospital discharge (Beggs 2015; Birkelbach 2019; Bissot et al. 2016). Consequently, older people living with frailty are at increased risk of perioperative morbidity.

Over the next 10 years the Office for National Statistics predicts a rise of 3.0 million in the UK’s overall population, with the number of people over 85 years expected to double in 25 years (Bock 2015). With frailty and ageing going hand-in-hand, it is likely we will see a rising number of frail patients presenting to hospitals for elective surgery. This growing and ageing population, combined with an increasing number of surgical procedures available, has led to unprecedented demand on the NHS for the provision of elective surgery. In England between 2014 and 2015, 2.5 million people aged over 75 years underwent surgery—a third of whom were aged over 85. This was a significant rise from just under 1.5 million between 2006 and 2007 (Bougeard et al. 2017). The intermittent suspension of elective operating during the COVID-19 pandemic has further increased demand, with over 5 million people now awaiting surgery in the UK.

In a recent survey of anaesthetic Perioperative Medicine leads across the UK, screening and management of frailty were identified as priorities to improve perioperative care (British 2017). Both screening and management, however, were recognised as being challenging to implement. Only 24% of respondents reported that their hospitals screened for frailty, and amongst those at least six different types of frailty assessment tools were in use (British 2017). The range of frailty assessment tools available vary in subjectivity, objectivity, ease of use, and on the model of frailty they assess. The Comprehensive Geriatric Assessment is the gold standard for the assessment of frailty but is impractical for routine use in the pre-operative setting. It requires over an hour to complete and includes assessments from multiple multi-disciplinary team members. The ideal tool for screening in the pre-operative setting would be time and staff-efficient, easy to perform, objective, and with high sensitivity and specificity. The Edmonton Frail Scale (EFS) has evidence for use in the perioperative setting (Care 2021) ( 2021) and is a tool supported by the British Geriatric Society (BGS) (Aucoin et al. 2020). It is quick to perform taking approximately five minutes to complete and, importantly, is able to assess multiple domains of frailty which may be subject to optimisation in the pre-operative period (Dhesi et al. 2019). Performing the physical assessment element of the EFS can be limiting in the pre-assessment setting. The Reported Edmonton Frail Scale (REFS) substitutes the observed “get up and go” assessment with a verbal report of physical function (Evered 2020; Grocott et al. 2017).

Despite its perceived importance, frailty is still not routinely screened for in all UK surgical patients. Its relevance in those attending for elective surgery therefore remains unclear. Our study, which we believe to be a novel study in the UK, aims to establish the prevalence of frailty in our local population during elective surgery. Additionally, we assessed whether frailty was associated with known peri-operative risk factors as well as length-of-stay and discharge status, thus evaluating its importance for patients during their perioperative journey. It is possible that better knowledge of prevalence would empower systematic changes in care pathways and subsequently improve outcomes for this high-risk group of patients.

## Methods

A prospective multi-centre observational cohort study was carried out in eight hospitals within the Wessex School of Anaesthesia between 1^st^ September and 30^th^ October 2020. Sites comprised a mixture of small and large district general hospitals and one tertiary centre. The study was undertaken in collaboration with the trainee-led South Coast Perioperative Audit and Research Collaborative (SPARC). Ethical approval was given by the South Central Hampshire B Research Ethics committee (20/SC/0121).

Patients were eligible for inclusion if they were aged 65 years or older and having elective surgery under general, regional or local anaesthesia. Patients were excluded if they declined to participate, were unable to give informed consent, were having emergency surgery or had difficulties with the English language. Patients were given an information sheet about the study on their arrival on the morning of surgery. Sufficient time was given for the patient to read the information sheet and to ask any questions. If they were happy to participate at this point written informed consent was obtained on the morning of surgery.

Each of the study sites collected data over three consecutive weekdays during the study period. The timing of the three days during the study period was at the discretion of local site leads. Participants were identified from departmental operating lists either on the day before or day of surgery. Basic demographics, co-morbidities and bloods were collected pre-operatively by anaesthetic trainees independent of the anaesthetic team delivering perioperative care. A second study team member blinded to the participant’s pre-operative dataset completed the REFS prior to surgery. All members collecting data had an up to date Good Clinical Practice (GCP) certification—a nationally recognised clinical research certification in the UK. Based on the REFS score (out of a maximum eighteen) participants were classified as Not Frail (0–5); Vulnerable (6–7); Mildly Frail (8–9); Moderately Frail (10–11); Severely Frail (12–18). Intra-operative and recovery data were collected once patients had returned to the ward or surgical day unit. Participants were followed-up at 30 days to assess length of stay, discharge status and location. Information contained within the REFS was recorded directly from the patient. All other relevant data was recorded from the patient's records and surgical pre-assessment forms.

Analyses were conducted on all patients for whom a frailty score was available and proceeded to surgery. Patients were registered on day of surgery and followed up at 30 days for outcomes. Data were summarised using means and standard deviations or percentages of categorical variables. The Kolmogorov–Smirnov test was used to assess for normality. Variations between people with and without frailty were estimated using two-sampled t tests or rank sum tests (non-parametric data) for differences between means or medians respectively, and chi-squared tests for correlation between categorical variables. A number of different outcomes were used to assess the impact of frailty on patients undergoing surgery, including length of stay, post-operative destination, mortality and discharge destination. Data were analysed using SPSS Statistics 26.0 (IBM Corp. Released 2019. IBM SPSS Statistics for Windows, Version 26.0. Armonk, NY: IBM Corp).

## Results

Data were collected on 228 patients during the study period. Ten patients did not proceed to surgery, therefore 218 patients were followed up at 30 days. Basic demographic data for these patients are summarised in Table 1.

**Table 1:** Participant demographics

All participant demographics (*n* = 218)	
**Age (years)**	
	75 (IQR 70–80)
**Gender**	
Female	103 (47.2%)
Male	115 (52.8%)
**BMI**	
	27 (IQR 24–30)
**Smoking status**	
Never smoked	108 (49.8%)
Ex-smoker	87 (40.0%)
Current smoker	12 (5.5%)
Unknown	10 (4.6%)
**Usual residence**	
Own home	211 (96.8%)
Sheltered accommodation	2 (0.9%)
Assisted living	1 (0.5%)
Family/friend	4 (1.8%)
**Carers**	
None	204 (93.6%)
Weekly	7 (3.2%)
Daily	4 (1.8%)
More than daily	3 (1.4%)
**Walking aids**	
Yes	61 (28.0%)
**Hearing aids**	
Yes	37 (17.1%)
**Visual aids**	
Yes	177 (81.6%)
**Cardiac history**	
No failure	107 (49.1%)
Diuretic, digoxin, antianginal or antihypertensive	91 (41.7%)
Oedema, warfarin or borderline cardiomegaly	16 (7.3%)
Raised JVP or cardiomegaly	2 (0.9%)
**Diabetes**	
No	175 (80.3%)
Type 2 (diet controlled)	24 (11.0%)
Type 2 (tablet controlled)	7 (3.2%)
Type 2 (on insulin)	11 (5.0%)
**ECG findings**	
No abnormalities	117 (53.7%)
AF rate 60–90	9 (4.1%)
AF rate > 90, paced or other dysrhythmia	35 (16.1%)

**Fig. 1:**
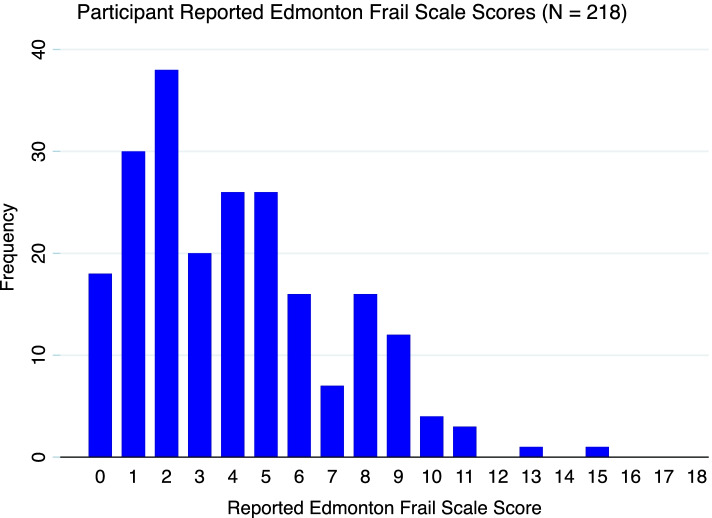
Distribution of Reported Edmonton Frail Scale scores (*n* = 218)

The median REFS score for all participants was 4 (IQR 2–6). The distribution of REFS scores is displayed in Fig. 1. Thirty-seven (17.0%) participants had a REFS score of eight or more and were classified as frail. The remaining 181 (83.0%) had a REFS score of seven or less and were classified as not frail or vulnerable. The breakdown of the number of participants in each frailty category by REFS score is summarised in Table 2. A large number of cases were day-case procedures, reflected in a median length-of-stay of zero days (IQR 0–3). The vast majority (95.0%) were discharged to their own homes.

**Table 2:** Reported Edmonton Frail Scale scores

All participants (*n* = 218)	
**Reported Edmonton Frail Scale outcome**	
Not frail (REFS less than 8)	181 (83.0%)
Frail (REFS 8 or more)	37 (17.0%)
**Frailty category**	
Not frail (REFS 0–5)	158 (72.5%)
Vulnerable (REFS 6–7)	23 (10.6%)
Mildly frail (REFS 8–9)	28 (12.8%)
Moderately frail (10–11)	7 (3.2%)
Severely frail (12–18)	2 (0.9%)
**Length of stay (days)**	
	0 (IQR 0–3)
**Discharged to own home**	
Yes	207 (95.0%)
No	7 (3.2%)
Not known	4 (1.8%)

Participants were recruited from a large number of surgical subspecialties, with orthopaedic, urological and ophthalmic procedures being the most commonly performed. The commonest ASA score was 2, though a third of participants did not have a pre-operative ASA score documented. The majority of participants had a general anaesthetic technique, most frequently using volatile anaesthetic agents. Important perioperative data are detailed in Table 3.

**Table 3:** Perioperative data

All participants (*n* = 218)	
**Surgical specialty**	
Orthopaedics	43 (19.7%)
Urology	37 (17.0%)
Eyes	22 (10.1%)
Abdominal – Lower GI	20 (9.2%)
Gynaecology	15 (6.9%)
Burns & Plastics	14 (6.4%)
Head & Neck	12 (5.5%)
Abdominal – Upper GI	9 (4.1%)
Cardiac	8 (3.7%)
Thoracic	7 (3.2%)
Vascular	7 (3.2%)
Neurosurgery	6 (2.8%)
Abdominal – Hepatobiliary	2 (0.9%)
Other	16 (7.3%)
**ASA**	
1	4 (1.8%)
2	75 (34.4%)
3	53 (24.3%)
4	6 (2.8%)
Not documented	80 (36.7%)
**Type of anaesthetic**	
General—inhalational	84 (38.5%)
General—target controlled infusion	51 (23.4%)
Local infiltration only	41 (18.8%)
Spinal	23 (10.6%)
Regional block	11 (5.0%)
Sedation	6 (2.8%)
Unknown	2 (0.9%)

Table 4 summarises the differences between frail (*n* = 37) and non-frail (*n* = 181) participants. In general, patients who were frail were more likely to be older (median age 78 years [IQR 74—86]) than patients who were vulnerable or not frail (median age 74 years [IQR 70–79], *p* < 0.001). There were no significant differences between frailty groups for other demographic variables such as gender (*p* = 0.102), smoking status (*p* = 0.095) and BMI (*p* = 0.297). Frail patients were more likely to have carers (*p* < 0.001) and use walking aids (*p* < 0.001). Overall, frail patients were more likely to have co-morbidities associated with poor surgical outcome than those who were not frail or vulnerable. Frail patients were more likely to be anaemic (62.1% vs. 32.6%, *p* = 0.003) and have multiple comorbidities as evidenced by higher ASA score (*p* < 0.001). Though not statistically significant there was a trend towards an increased prevalence of diabetes in frail patients (29.7% vs. 17.2%, *p* = 0.079) and similarly, frail patients were more likely to present with a history of cardiac disease (74.3% vs. 45.9%, *p* = 0.002) and abnormal ECG findings (38.5% vs. 18.5%], *p* = 0.024).

**Table 4:** Comparison between frail and non-frail patients

	**Not frail (*n* = 181)**	**Frail (*n* = 37)**	***p*-value**
**Age (years)**			
	74 (IQR 70–79)	78 (IQR 74–86)	** < 0.001**
**Gender**			
Female	81 (44.8%)	22 (59.5%)	0.102
Male	100 (55.2%)	15 (40.5%)	
**BMI**			
	27.0 (IQR 24.0–30.4)	26.0 (IQR 24.1–29.4)	0.297
**Smoking status**			
Never/Ex	167 (95.4%	28 (87.5%)	0.095
Current	8 (4.6%)	4 (12.5%)	
**Any carers**			
No carers	176 (97.2%)	28 (75.7%)	** < 0.001**
Carers	5 (2.8%)	9 (24.3%)	
**Walking aids**			
Yes	39 (21.5%)	22 (59.5%)	** < 0.001**
**Hearing aids**			
Yes	28 (15.6%)	9 (24.3%)	0.196
**Visual aids**			
Yes	145 (80.6%)	32 (86.5%)	0.397
**Cardiac disease**			
Yes	83 (45.9%)	26 (74.3%)	**0.002**
**Diabetes**			
Non-diabetic	149 (82.8%)	26 (70.3%)	0.079
Diabetic	31 (17.2%)	11 (29.7%)	
**ECG abnormalities**			
Normal or rate controlled AF	110 (81.5%)	16 (61.5%)	**0.024**
AF rate > 90, paced or other dysrhythmia	25 (18.5%)	10 (38.5%)	
**ASA score**			
Not documented	68 (37.6%)	12 (32.4%)	** < 0.001**
1	4 (2.2%)	-	
2	70 (38.7%)	5 (13.5%)	
3	35 (19.4%)	18 (48.7%)	
4	4 (2.2%)	2 (5.4%)	
**Haemoglobin (g/L)**^**a**^			
	135.1 (± 14.8)	126.4 (± 14.8)	**0.005**
**Anaemia (Haemoglobin < 130 g/L)**^**a**^			
Yes	43/132 (32.6%)	18/29 (62.1%)	**0.003**
**Length of stay (days)**			
	0 (IQR 0–2)	0 (IQR 0–4)	0.681
**Discharged to own home**			
No	1 (0.6%)	6 (17.1%)	** < 0.001**
Yes	178 (99.4%)	29 (82.9%)	

^a^ Data available for 161 participants

The mortality rate was less than 1% for the entirely study population. However, the one patient who passed away was frail according to REFS. Similarly, four patients were still in hospital at the end of the study period, two frail and two not frail. Length of stay did not differ between frail and non-frail participants (*p*-value = 0.681). Discharge destination was significantly different (*p*-value < 0.001), with frail patients more likely to be discharged with family and friends, into sheltered accommodation or to other destinations not their own home. However, the total number of cases in each of these groups was very small, as 29 [82.9%] frail patients were discharged to their own home compared to 178 [99.4%] not frail patients.

## Discussion

In this prospective observational study of patients aged 65 or older undergoing elective surgery, we established the prevalence of frailty to be 17.0% using the Reported Edmonton Frail scale. Frail patients were more likely to present with modifiable pre-operative co-morbidities, require carers, and were less likely to be discharged to their own homes following surgery. All of these are relevant factors in the planning of personalised perioperative care.

To our knowledge, this is the first study to assess the prevalence of frailty in patients undergoing solely elective surgery in the UK. The prevalence of frailty in the surgical population varies widely in the literature and is influenced by both the tool being used and the population being assessed. The largest study to date of frailty in patients undergoing elective and emergency surgery (over 430,000 American veterans) found 8.5% patients to be frail (He et al. 2020) whereas a recent meta-analysis of over 2000 general surgical patients (elective and emergency) estimated the prevalence to be higher, at between 10 and 37% (Hewitt et al. 2018). Few studies have been undertaken in the UK and all are a mix of elective and emergency patients, for example a study of emergency and elective vascular patients in a UK setting found 52% of patients aged over 60 were frail, using the Edmonton Frail Scale (Hilmer et al. 2009). The use of the REFS as our frailty assessment tool has potential limitations. Patient reporting of functional status has a subjective element in its assessment instead of the timed “up and go” test it replaces, though it’s use has been validated in previous studies (Evered 2020; Makary et al. 2010). This tool needed to be used in a variety of different pre-operative settings across multiple hospitals. There was the potential difficulty of reliably reproducing the timed “up and go” test across multiple sites during the Covid-19 pandemic, therefore REFS was chosen.

One of the key findings of our study was that many frail patients had reached the day of planned surgery with medical problems which, had they been identified earlier, could have been corrected or optimised in advance. For example, nearly two thirds of frail patients were found to be anaemic compared to only a third of non-frail patients. Identifying and treating these frail patients, who appear to have a significantly higher risk of anaemia, could reduce associated post-operative complications (He et al. 2020). There was also a trend towards frail patients having a higher rate of diabetes, another important perioperative risk factor. The use of a frailty screening tool such as the REFS could highlight these patients earlier in the perioperative pathway, allowing time for pre-operative optimisation and a reduction in post-operative complications (National 2018; Oakland et al. 2016). The Royal College of Anaesthetists emphasises that frailty requires a cross-specialty approach to enable optimisation of medicines and improved management of non-surgical comorbidities. This has been consolidated by the recent ‘Guideline for Perioperative Care for people living with frailty undergoing Elective and Emergency surgery’ by the Centre of Perioperative Care and British Geriatric Society (Partridge et al. 2015). A challenge is that to achieve this requires both time and resources, especially when considering the impact of Covid-19 on elective surgery (Robinson et al. 2009). Whilst the REFS was easily implemented on the morning of surgery, clearly screening would be best performed earlier in the perioperative pathway to allow time for intervention, for example at time of referral from primary care. A practical alternative could be to integrate the REFS (or other frailty assessment tool) into the routine pre-operative assessment questionnaires already performed by hospital pre-admission teams.

Knowledge of the presence and severity of frailty can help with assessing surgical risk (Robinson et al. 2011) and, thus, help inform shared decision-making discussions and ensure validity of consent (Rolfson et al. 2006). Having been informed of their higher-risk status, some patients may choose to proceed with planned surgery, whilst others may elect for an alternative procedure or choose not to have surgery at all (Shinall 2020). Advanced knowledge of frailty can help ensure appropriate personnel and equipment are available in theatres on the day of surgery. For example, frail patients may be given a longer time-slot for induction of anaesthesia, or be cared for by a more senior anaesthetist. There may be adjustments that need to be made to the anaesthetic technique; such as additional monitoring or selection of regional blockade in place of general anaesthesia. Discussion of these interventions in advance of surgery will also allow patient expectations to be managed. This highlights the need for early pre-assessment to allow adequate time for potential interventions, reflected in recent considerations to radicalise the patient pathways before surgery (Skaar et al. 2021).

From the point-of-view of post-operative care and discharge planning, an awareness of frailty pre-operatively can also ensure adequate planning and resource allocation. For example frail patients could be identified in advance of surgery so that physiotherapists, dietitians and a clinician with an interest in perioperative medicine for the elderly could be involved in planning their post-operative care. Not only could this improve quality of care but may also optimise hospital efficiency through reduced length-of-stay and cancellations. Although our study did not demonstrate significant differences in length-of-stay we did show that 99% of non-frail patients were discharged to their own home, compared to only 83% of frail patients. This again may suggest a benefit for discharge planning in identifying these patients early on in their surgical journey.

One of the main strengths of this study was that it recruited participants from a wide geographical area incorporating a mixture of small and large hospitals serving a mixture of inner city, semi-rural and rural communities. In practice we found the REFS questionnaire to be quick to perform; taking only five minutes or less to complete. It was also found to be acceptable to patients. The junior doctors administering the questionnaire, predominantly anaesthetic trainees, did not require any additional training which suggests that the REFS would be quick and cost-effective to introduce into routine clinical practice. The delivery of a multi-centre study by doctors in training is another strength.

However, this study has limitations. The participants were recruited from a heterogeneous group of surgical specialties and it is likely that different specialties will have differing rates of frailty. Furthermore, the COVID-19 pandemic impacted on elective surgery to an extent still difficult to ascertain. While this study was conducted during a period of relatively increased operating activity, it is likely that patients listed for surgery were selected because they were from a less vulnerable patient group or not shielding, this may have led to pre-selection bias. The pandemic also led to a shortage of high dependency and intensive care beds, meaning fewer high-risk elective procedures were performed (Activity 2006). A further factor affecting the study’s generalisability was that patient’s without capacity were excluded. Some of the most severely frail patients presenting for surgery, for example with advanced dementia, will therefore not have been approached.

## Conclusions

In conclusion, we found the prevalence of frailty in a mixed population of elective surgical patients aged 65 or over to be 17.0%. Furthermore, we found the REFS to be a practical tool for pre-operative frailty screening. Frail patients presented more frequently with modifiable co-morbidities, such as anaemia, which could have been optimised pre-operatively. Adequately powered future studies should further assess the impact of frailty, and we look forward to the results of the upcoming third Sprint National Audit Project (Tao et al. 2008). Early screening for frailty could highlight frail patients and allow time for evidence-based pre-operative planning and interventions to be made, and we therefore encourage frailty assessment to be a routine part of pre-operative assessment with the early involvement of relevant healthcare professionals. With waiting times for elective surgery at an unprecedented high, we have both an opportunity and responsibility to use this time well.

## Data Availability

The datasets used and/or analysed during the current study are available from the corresponding author on reasonable request.

## Abbreviations

ASA: American Society of Anaesthesiologists
BMI: Body Mass Index
COVID-19: Severe Acute Respiratory Syndrome Coronavirus 2
ECG: Electrocardiogram
EFS: Edmonton Frail Scale
HRA: Health Research Authority
IQR: Interquartile Range
REFS: Reported Edmonton Frail Scale
SD: Standard deviation
SPARC: South Coast Perioperative Audit and Research Collaborative
UK: United Kingdom
